# Repetitive Robot Behavior Impacts Perception of Intentionality and Gaze-Related Attentional Orienting

**DOI:** 10.3389/frobt.2020.565825

**Published:** 2020-11-09

**Authors:** Abdulaziz Abubshait, Agnieszka Wykowska

**Affiliations:** Social Cognition in Human-Robot Interaction (S4HRI) Unit, Istituto Italiano di Tecnologia, Genova, Italy

**Keywords:** social cognition, human robot interaction, gaze cueing, intentional stance, intention attribution, attention orienting

## Abstract

Gaze behavior is an important social signal between humans as it communicates locations of interest. People typically orient their attention to where others look as this informs about others' intentions and future actions. Studies have shown that humans can engage in similar gaze behavior with robots but presumably more so when they adopt the intentional stance toward them (i.e., believing robot behaviors are intentional). In laboratory settings, the phenomenon of attending toward the direction of others' gaze has been examined with the use of the gaze-cueing paradigm. While the gaze-cueing paradigm has been successful in investigating the relationship between adopting the intentional stance toward robots and attention orienting to gaze cues, it is unclear if the repetitiveness of the gaze-cueing paradigm influences adopting the intentional stance. Here, we examined if the duration of exposure to repetitive robot gaze behavior in a gaze-cueing task has a negative impact on subjective attribution of intentionality. Participants performed a short, medium, or long face-to-face gaze-cueing paradigm with an embodied robot while subjective ratings were collected pre and post the interaction. Results show that participants in the long exposure condition had the smallest change in their intention attribution scores, if any, while those in the short exposure condition had a positive change in their intention attribution, indicating that participants attributed more intention to the robot after short interactions. The results also show that attention orienting to robot gaze-cues was positively related to how much intention was attributed to the robot, but this relationship became more negative as the length of exposure increased. In contrast to subjective ratings, the gaze-cueing effects (GCEs) increased as a function of the duration of exposure to repetitive behavior. The data suggest a tradeoff between the desired number of trials needed for observing various mechanisms of social cognition, such as GCEs, and the likelihood of adopting the intentional stance toward a robot.

## Introduction

Humans rely on non-verbal behavior to interact with one another. For example, when we observing someone looking at an apple, we infer that that person is hungry. This process, in which people make inferences about what others are thinking, is called mentalizing (Baron-Cohen, 1995), and is essential for successful human social interactions (Frith and Frith, 2006). Mentalizing is a highly automatic process that we engage in (Frith and Frith, 2006). Assuming that others' behavior is based on their thoughts, emotions, and internal states is called adopting the intentional stance (Dennett and Haugeland, 1987; Dennett, 1989). While it is assumed that when humans interact with one another they adopt the intentional stance (i.e., they explain and predict others' behaviors with reference to their underlying thoughts, feelings, and intentions), an interesting question remains if (and when) people adopt the intentional stance toward artificial agents that resemble humans, such as humanoid robots. Although prior studies (e.g., Marchesi et al., 2019) have uncovered instances where people do adopt the intentional stance toward humanoid robots, here we ask if certain procedures used in experimental design can actually create obstacles for measuring the intentional stance in human–robot interaction (HRI).

Several studies do in fact show that people are capable of adopting the intentional stance toward artificial agents in some cases. For example, when robots, avatars, or computers perform unexpected actions (Morewedge, 2009; Waytz et al., 2010), resemble humans in their physical appearance (Kiesler et al., 2008; Admoni et al., 2011), display emotions (Fussell et al., 2008), react to people's actions (Terada et al., 2007), exhibit humanlike non-verbal behaviors such as shrugging (Carter et al., 2014), cheat (Short et al., 2010), or engage in eye contact with others (Ito et al., 2004; Yonezawa et al., 2007), humans are more likely to adopt the intentional stance toward them. When humans adopt the intentional stance, it can lead to positive outcomes in HRI^1^ as robots that are perceived as intentional are seen as more trustworthy (Kiesler et al., 2008; Carter et al., 2014), are able to engage humans better (Yamazaki et al., 2010), and have positive effects on people's moods (Carter et al., 2014). Adopting the intentional stance toward robots can also have positive effects on human performance in HRI as they can facilitate learning (Brown and Howard, 2013), improve physical interactions where hand–eye coordination is needed (Carter et al., 2014), induce social facilitation effects (Bartneck, 2003; Woods et al., 2005; Looije et al., 2010), and improve interactions in team settings (Breazeal et al., 2005). By the same token, not adopting the intentional stance toward social robots can pose a problem for HRI, as humans might be engaging fewer socio-cognitive mechanisms in social interactions with robots, and thereby less social attunement (i.e., activation of socio-cognitive mechanisms) occurs between the two agents (Wiese et al., 2012, 2018; Wykowska et al., 2014; Özdem et al., 2016; Caruana et al., 2017; Ciardo et al., 2020; for a review, see Perez-Osorio et al., 2015; Wiese et al., 2017; Schellen and Wykowska, 2019).

One successful paradigm to measure social attunement in HRI is the gaze-cueing paradigm (Wiese et al., 2012; Wykowska et al., 2014; Perez-Osorio et al., 2015; Kompatsiari et al., 2018) as prior work has shown that attentional orienting to gaze cues is the foundation of many (often higher-level) social-cognitive processes, such as mentalizing (Baron-Cohen, 1995; Nummenmaa and Calder, 2009; Teufel et al., 2010; Pfeiffer et al., 2011). The gaze-cueing paradigm generally starts with a gazing stimulus (i.e., a face or face-like drawing) that looks directly at an observer (i.e., participant) and then makes a gaze shift to a certain direction (e.g., right or left), which shifts the observer's attention to the gazed-at location (Friesen and Kingstone, 1998; Driver et al., 1999; Langton and Bruce, 1999; Quadflieg et al., 2004), if the observer engages in joint attention with the gazer. After the gaze- shift, a target appears either in the same (i.e., valid trial) or a different location (i.e., invalid trail) relative to the gaze cue. Studies have consistently shown that people are faster at responding to valid trials compared to invalid trials, with the magnitude of the difference between valid reaction times and invalid reaction times indicative of how strongly or how frequently attention was oriented to the gazed-at location (i.e., the gaze-cueing effect: GCE). Studies investigating the GCE have also shown that the effects are observed even when gaze was counter-indicative of the target location, which suggests the reflexivity of attention orienting to gaze cues (Ristic and Kingstone, 2005; Kingstone et al., 2019).

More recent studies have shown that attention orienting to gaze cues can be top-down modulated (i.e., volitional), depending on the social relevance of the gaze. For example, attention orienting to gaze cues is stronger when the gazer is of higher social rank (Shepherd et al., 2006; Jones et al., 2010; Ohlsen et al., 2013; Cui et al., 2014; Dalmaso et al., 2014), is physically similar to the observer (Hungr and Hunt, 2012), is perceived as trustworthy (Süßenbach and Schönbrodt, 2014), has the possibility to see (Teufel et al., 2010), or when gaze cues are meaningful to the context of the gaze (Perez-Osorio et al., 2015). Paramount to our study, prior work has also shown that GCEs are enhanced when gaze shifts are thought to originate from an intentional agent (Wiese et al., 2012; Wykowska et al., 2014; Caruana et al., 2017), compared to just a pre-programmed machine, which suggests that adopting the intentional stance toward a robot can influence how strongly it can induce attentional orienting to its directional cues.

Although previous literature showed that, indeed, people are able to socially attune with a robot, and perhaps more strongly so if they adopt the intentional stance toward it, more recent studies have shown that using artificial agents in gaze-cueing studies can have negative consequences (Abubshait et al., 2020a). Therefore, it is unclear if classical psychological paradigms such as the gaze-cueing paradigm are the best choice to investigate socio-cognitive mechanisms, due to the fact that the gaze-cueing paradigm is quite unnatural in terms of social context. Not only does it often employ 2D stimuli on the screen (which is unlike natural social interactions), but it also uses repetitive movements (i.e., the gaze-cueing paradigm employs many trials of robots gazing in one of two directions). Prior work has shown negative impacts of multiple exposures on subjective perceptions of robots over time (Bergmann et al., 2012), and recent approaches in social neuroscience (Schilbach et al., 2013) highlight the need for more ecologically valid paradigms for studying mechanisms of *social* cognition. Furthermore, the fact that multiple interactions over time might change our perceptions of a robot is in line with Epley et al's (2007) theory of anthropomorphism, which suggests that repetitive interactions can decrease our likelihood to attribute mental states (i.e., adopting the intentional stance) toward a robot since we no longer have the motivation to understand its behavior: *effectance motivation*. While prior studies have shown negative impacts of multiple exposures on subjective ratings, it is unclear if this effect can also be generalized to behavioral measurements, such as the GCEs. Specifically, it is unclear if the standard procedure of the gaze-cueing paradigm in HRI conceals aspects of social attunement, due to the fact that many trials are needed to establish an accurate and stable GCE (e.g., face-to-face studies include 160 trials; Lachat et al., 2012).

In the current study, we examined the effect of the duration of exposure to repetitive gaze behavior on both subjective perceptions of intentionality and gaze-induced attentional orienting in a gaze-cuing paradigm. Using a face-to-face gaze-cueing paradigm with a humanoid robot (i.e., iCub: Metta et al., 2008), participants were assigned to either a *short exposure* (i.e., 96 trials), a *medium exposure* (i.e., 176 trials), or a *long exposure* condition (i.e., 256 trials), while we collected pre- and post-ratings of adopting the intentional stance (InStance Questionnaire, Marchesi et al., 2019) and attributing mental capacities (Weisman et al., 2017). If the duration of interaction with a robot does indeed influence attribution of intentionality toward a robot, we expect that participants assigned to the different exposure conditions would experience changes in likelihood to adopt the intentional stance toward iCub after their interaction differently. To that extent, we expected to observe a different impact of interaction with the robot on the adoption of the intentional stance, depending on duration of the interaction. Additionally, we hypothesized that the relationship between adopting the intentional stance and GCEs (i.e., social attunement via attention orienting) would depend on the duration of exposure to repetitive behavior.

## Methods

### Participants

Twenty-seven participants were recruited (M = 24.4; range = 19–49; 17 females; 25 right-handed) and were quasi-randomly assigned to one of three exposure conditions (i.e., *short, medium*, or *long exposure*). Participants were compensated 15 euros upon completion of the study. The experiment was conducted in accordance with the World Medical Association Declaration of Helsinki ethical principles for medical research involving human subjects and was approved by the local Ethical Committee (Comitato Etico Regione Liguria). An a priori power analysis was conducted using R version 3.6 and the pwr package to determine our sample size. The analysis was based on an f test of three independent groups, an alpha of 0.05, a medium effect size of 0.4 (based on prior research: Kompatsiari et al., 2018) and power set to (1—b = 0.8). The analysis resulted in a sample size of 63 participants (i.e., 21 participants per group). While the a priori power analysis suggested a larger sample (*N* = 63), due to nationwide COVID-19 lockdowns, we were only able to collect half of the required sample. To alleviate this issue, we used Bayesian models to test our hypotheses as opposed to parametric analyses. While Bayesian inferences are not immune to symptoms related to small sizes, the use of the correct priors can provide an advantage to testing our hypotheses (McNeish, 2016).

### Apparatus

The gaze-cueing experiment used the iCub robot along with two 27-in. screens that were rotated sideways (i.e., vertically) for target presentation (see Kompatsiari et al., 2018 for a similar setup). iCub's movements were controlled by the YARP gaze platform (Metta et al., 2006). iCub's eye vergence was set to 5° and remained constant, while the timing trajectories of the eye and neck movements were set to 200 and 400 ms, respectively, to allow for smoother and natural-looking movements. Data collection and target presentation were controlled and programmed using OpenSesame (Mathôt et al., 2012). Responses were collected using a standard keyboard.

### Questionnaires

The questionnaires used in the study included the Intentional Stance questionnaire measuring people's tendencies to adopt the intentional stance toward robots (Marchesi et al., 2019); the anthropomorphism questionnaire measuring people's anthropomorphic and attachment tendencies (Neave et al., 2015); the Body, Heart and Mind questionnaire measuring people's perceptions of mental life (Weisman et al., 2017); and finally a simple perceived predictivity question measuring how much subjects can predict iCub's actions. The gaze-cueing task also included a question regarding people's subjective perceptions of their engagement toward iCub (see gaze-cueing task section for details). All items were translated and administered in Italian and can be found on osf.io/y6c9b.

The Intentional Stance Questionnaire is a set of 34 scenarios. Each scenario contained three pictures of iCub involved in regular daily scenes (e.g., playing cards with another human, sorting objects into containers). Below each scenario, two sentences were presented that described the scenario, one in mentalistic terms (e.g., “iCub pretends to be a gardener”) presented on the right, and the other in mechanistic terms (e.g., “iCub adjusts the force to the weight of the object”) presented on the left. Participants were tasked to move a slider (positioned initially between the two sentences) toward the description that best fit the scenario. The logic behind this questionnaire was that if participants moved the slider toward the mentalistic description, they likely adopted the intentional stance, while if they chose the mechanistic description, they were more likely to adopt the “design stance” (Dennett and Haugeland, 1987) for explaining the robot's behavior. Based on a split-halves analysis, the questionnaire was split into two sections that were correlated with each other. The first half was presented at the beginning of the study, and the second half was presented at the end of the study, with the order of items of each half presented randomly. The items were scored on a 10-point-magnitude scale with an additional decimal point, with 0 indicating adopting the design stance and 10 indicating adopting the intentional stance. The scores were calculated by averaging all items together. Descriptions of the remaining questionnaires can be found in the Supplementary Material.

### Gaze-Cueing Protocol

**Figure 1:**
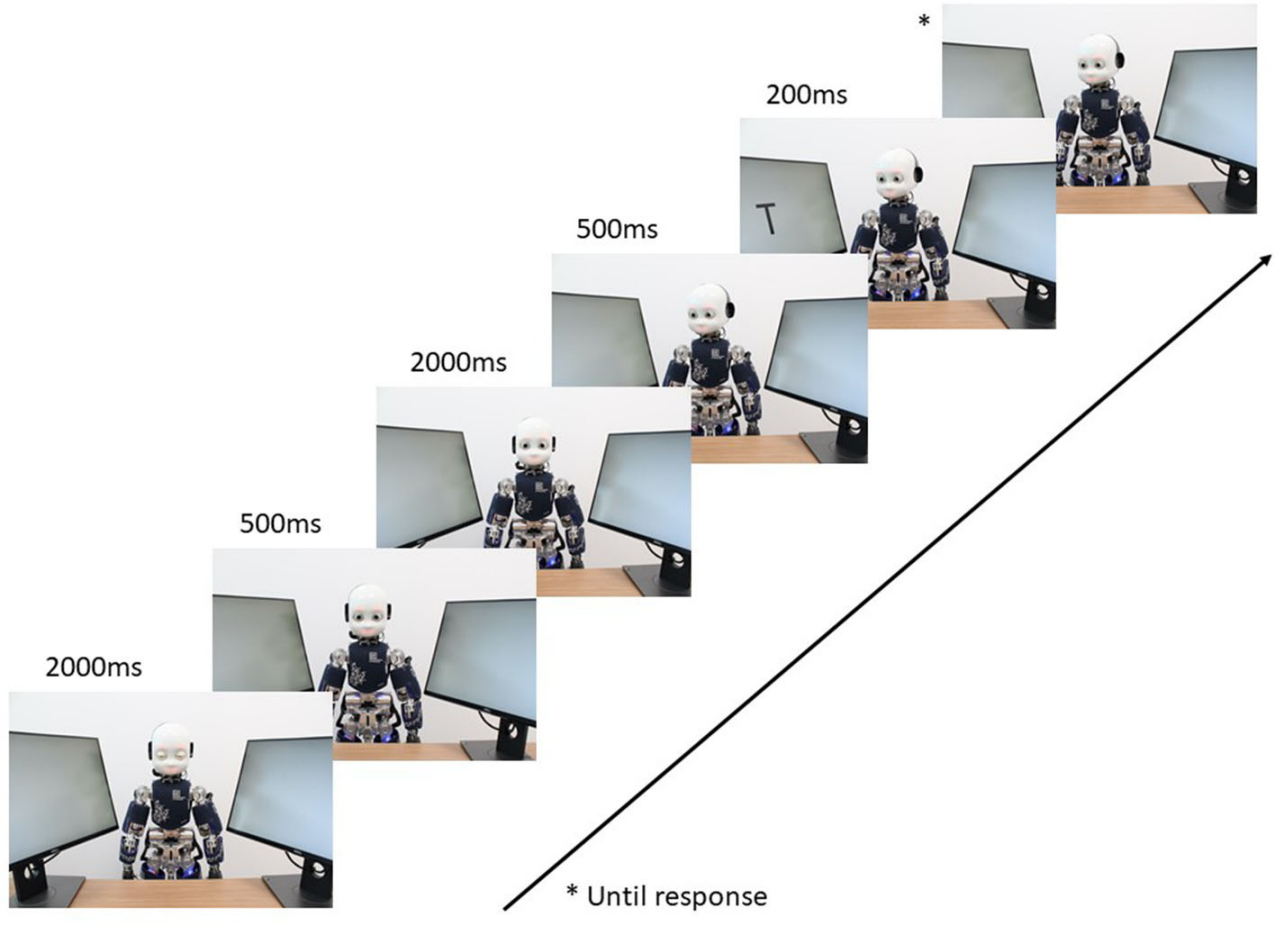
Trial sequence of the gaze-cueing paradigm: Each trial started with iCub's eyes closed. iCub would then open its eyes and fixate on the observer's eyes to engage in mutual gaze. Next, iCub would make a gaze shift to either the right or left. Next, a target appeared for 200 ms. If the target appeared in the same direction of the cue, the trial was considered valid. Targets appeared in the opposite direction of the cue for invalid trials. The stimulus onset asynchrony (SOA) was set to 500 ms.

Each trial of the gaze-cueing paradigm started with iCub's eyes closed. 2,000 ms later, iCub opened its eyes and looked directly at the participant for 500 ms; 2,000 ms later, iCub looked at one of the two screens (i.e., at the left screen or the right screen); 500 ms later, a target (i.e., a T or V) appeared on either of the two screens. On valid trials, the target appeared on the same screen that iCub was looking at. On invalid trials, the target appeared on the opposite screen (e.g., iCub looked at the right screen and the target appeared on the left screen). The target appeared for a duration of 200 ms, and iCub remained looking at the screen until participants responded, which ended the trial. iCub's gaze validity was non-predictive of the target location (i.e., 50% valid trials and 50% invalid trials). Each block consisted of 16 trials, which ended by asking participants about their subjective perception of iCub's engagement. The *short exposure* condition consisted of 96 trials (i.e., 6 blocks), the *medium exposure* condition consisted of 176 trials (i.e., 11 blocks), and the *long exposure* condition consisted of 256 trials (i.e., 16 blocks). See Figure 1 for the trial sequence of the Gaze-cueing protocol.

### Procedure

After participants provided written consent, they were seated at a desk ~125 cm away from iCub. The two screens were placed laterally on the desk 32.5 cm away from the robot on each side (i.e., 75 cm apart from one another). The screens were tilted back vertically 12.5° and rotated 76° toward the participant from the lateral position. The screens were ∽105 cm away from the participants. The target stimuli were either a “T” or “V” and were 4.5° and 7°.

Next, participants completed the first set of questionnaires, which included an anthropomorphism questionnaire (Neave et al., 2015), the Intentional Stance Questionnaire (ISQ, Marchesi et al., 2019), a mental capacity questionnaire (Weisman et al., 2017), and a perceived predictivity question. After completing the questionnaires, which were presented in random order, participants completed a practice session of the gaze-cueing task with iCub. The practice session contained 16 gaze-cueing trials. One half of participants were instructed to press the “T” key with their right index finger and the “V” key with their left index finger, while the other half were instructed to do the opposite. Next, participants completed the experimental gaze-cueing task. Upon completion, participants moved on to the post-questionnaire phase, which included the ISQ, the mental capacity questionnaire, and the predictivity question. Finally, participants were debriefed and thanked for their participation.

### Analyses

#### Main Analyses

While classical parametric and null hypothesis significance testing (NHST) require larger sample sizes, we opted to use Bayesian inference to alleviate issues with the inability to collect the desired sample size. Bayesian inference can provide three major advantages that directly influence the present study. First, we alleviate issues that are related to smaller sample sizes due to the fact that the use of prior distributions to estimate the posterior distributions can help find better estimates of the relationships in question (McNeish, 2016). Second, using Bayesian inference provides the advantage of examining the results in terms of probabilities, and not simply whether the null hypothesis is accepted or rejected, which is easily interpreted and can be generalized to future studies. Finally, we are able to use credible intervals that fit our data and not simply ones that are conventional, as researchers suggest that the conventional 95% credible interval band is not appropriate for Bayesian statistics due to the fact that it restricts our posterior distributions (Kruschke, 2014). While some researchers suggest using an 89% credible interval (McElreath, 2020), we opted to use a 90% credible interval for rounding purposes. For both Bayesian models, we used five Markov Chain Monte Carlo (MCMC) chains with 10,000 samples. The first 500 samples were used as warm-up and discarded. All Bayesian models used weakly informed priors based on the observed mean and a wide SD (i.e., SD = 10).

To measure changes in questionnaire scores, we first scored the pre-items and post-items separately. Next, we calculated a difference score between pre- and post-scores (i.e., post-score—pre-score). Positive values would indicate higher scores after exposure. Negative values would indicate lower scores after exposure. A value of 0 would suggest no change after exposure. By creating difference scores, we were able to examine how participants changed their scores as opposed to only predicting their final scores. To examine whether people adopted a different stance after exposure, we used a Bayesian regression model to predict participants' difference ISQ scores from the exposure length (a dummy coded variable: Short = 0, Medium = 1, Long = 2) and each participant's Pre-ISQ scores. No interaction term was included between the two variables as we were only interested in the effect of exposure duration. Using this method would allow us to compare each of our conditions to the Short Exposure condition without the need for follow-up tests and control for baseline differences in ISQ scores. Mean ISQ scores for the three exposure duration groups are presented in Table 1.

**Table 1:** Instance questionnaire scores.

	**Pre ISQ**		**Post ISQ**		**Difference ISQ**	
**Exposure Length**	**M**	**SD**	**M**	**SD**	**M**	**SD**
Short	4.20	2.96	4.77	2.60	0.57	1.27
Medium	3.18	2.11	3.76	1.28	0.58	1.12
Long	3.98	1.66	4.06	0.08	0.08	1.18

*Positive ISQ differences indicates a higher rating at post*.

To examine if adopting the intentional stance affected GCEs as a function of Exposure, we first calculated a GCE for each participant. To do so, we excluded trials that were faster than 100 ms and slower than 1,500 ms. Next, we removed incorrect trials and trials that were slower than 2.5 SD away from the individual mean. Afterwards, we calculated the GCE, which is the difference between valid and invalid reaction times (i.e., invalid RT—valid RT), with larger positive GCE indicating stronger attention orienting in relation to iCub's gaze. After calculating the GCE, we used a Bayesian regression to regress GCE onto the mean-centered Pre-ISQ scores, Exposure length (i.e., a dummy coded variable: Short = 1, Medium = 2, Long = 3), and their interaction. This regression model would allow us to examine (A) if gaze-cueing effects were different depending on the length of exposure to repetitive behavior (i.e., main effects) and (B) if the relationship between GCEs and ISQ scores is dependent on exposure length. Similar to the previous analysis, using dummy coding allows us to follow up on any interactions without the use of *post-hoc* tests. Mean RTs in valid and invalid conditions across the three exposure duration groups are presented in Table 2.

**Table 2:** Reaction time data.

	**Valid**		**Invalid**		**GCE**	
**Exposure Length**	**M**	**SD**	**M**	**SD**	**M**	**SD**
Short	494.99	59.18	502.64	68.00	7.65	11.80
Medium	484.23	125.46	496.86	118.06	12.62	13.86
Long	477.23	50.63	492.49	56.33	15.26	10.19

*GCE is the difference between valid and invalid trials*.

#### Exploratory Analyses

Additional exploratory analyses were conducted to investigate if attribution of mental capacities (i.e., Body, Heart, and Mind; Weisman et al., 2017) and perceived predictivity were correlated with gaze-cueing effects, if individual differences in anthropomorphism were correlated to adopting the intentional stance, how adopting the intentional stance related to attribution of mental capacities, if the perceived predictivity and mental capacity ratings changed as a function of exposure, and if subjective ratings of engagement decreased over time, as prior work has shown a correlation between robots that engage in gaze behavior and subjective ratings of engagement (Admoni et al., 2011; Kompatsiari et al., 2018). Due to the exploratory nature of these analyses, we only report descriptive statistics, coefficient estimates, and the 90% confidence level rather than significance level, as not to inflate alpha levels. Exploratory analyses and results can be found in the Supplementary Material.

## Results

### Main Results

All participants performed the gaze-cueing task with high accuracy (M = 95.8%, SD = 0.03). One participant was identified as an outlier and was removed from the analyses due to a GCE score of more than 3 SD away from the mean (SD = −3.19). Data of the remaining 26 participants were analyzed.

#### InStance Scores

**Figure 2:**
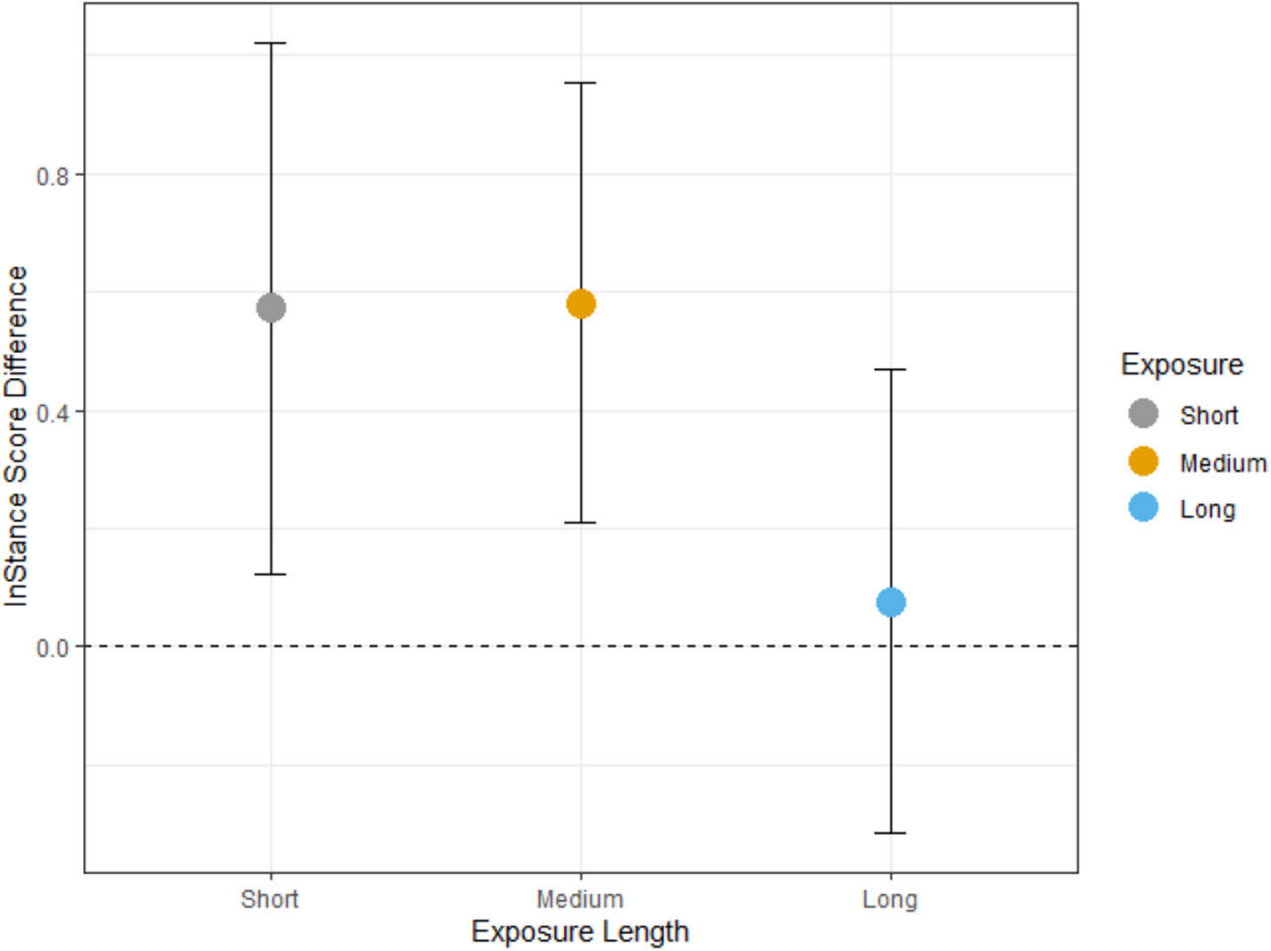
Change in ISQ ratings after exposure. The graph illustrates the difference in pre- and post-ratings. A positive increase illustrates that the rating changed to a higher rating at post, while a zero value would indicate no changes between pre- and post-ratings. The observed ISQ difference scores show the short and medium exposure conditions show a positive increase in ISQ scores after completing of the gaze-cueing task, while the long exposure condition shows no increase.

The Bayesian model did not run into issues with convergence and fit the observed data well ($\hat{\text{R}}$ = 1 and MCSE = 0 for all parameters). It took about 0.92 s for each chain to converge. The posterior distribution of the Bayesian model showed that the short exposure condition had a mean of 1.87 with a 90% PI [1.01, 2.72], the medium condition had a mean of 1.57 with 90% PI [0.77, 2.39], and the long condition had a mean of 1.32 with 90% PI [0.53, 2.12]. When examining the means, the 90% credible intervals of the posterior sample show that there is a 27% chance that the short and medium conditions overlap in their estimates, which suggests that there is a meaningful difference in their estimates. This difference is even smaller for the short and long conditions as there is a 12% chance that the means overlap. In other words, there is a 27 and a 12% chance, respectively, that the medium exposure and long exposure conditions contain a mean of 1.87, respectively. The model also predicts that there is an 83% overlap between the 90% credible intervals of the short and medium posterior distributions and a 70% overlap between the short and long distributions. This suggests that there is a 17% chance that a true difference between short and medium conditions exists, and a 30% chance that a true difference between the short and long conditions exists. See Figure 2 for the observed data and Supplementary Figure 1A for the posterior distributions. This suggests a linear relationship between exposure length and adopting the intentional stance.

Due to a clear descriptive difference between the medium exposure condition and the long exposure condition in Table 1 and Figure 2, we also examined the probabilities that the two means overlap. The posterior distributions showed that there is a 30% chance that the means of the medium exposure condition and the long exposure condition overlap. In other words, there is a 70% chance that there is a true difference between the two means. This additional test provides more evidence for the linearity of the relationship between exposure duration and the change in ISQ ratings.

#### Gaze Cueing Effects

**Figure 3:**
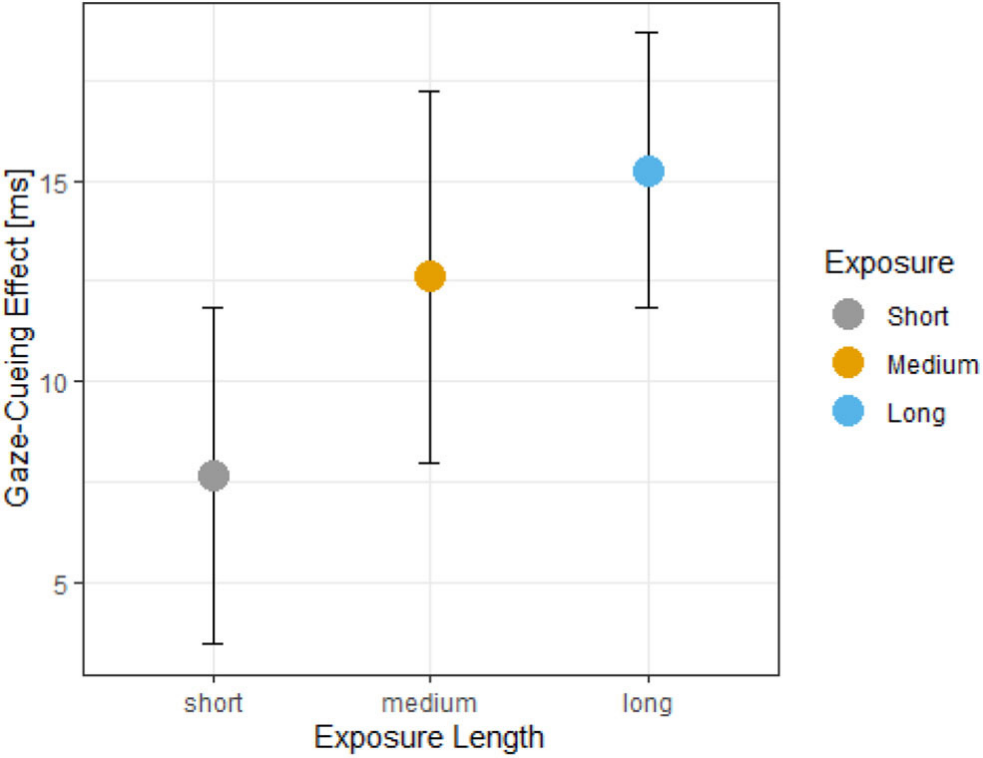
Gaze-cueing effects (GCEs) as a function of exposure. The graph illustrates the mean values of GCE, which is the calculated difference between valid and invalid trials, with more positive values indicating stronger attention orienting in response to iCub's gaze shifts. The GCEs show that attention orienting to gaze-cues is enhanced as the length of exposure (i.e., trial numbers) increased.

The Bayesian model predicting GCEs did not have issues with convergence ($\hat{\text{R}}$ = 1, MCSE_Medium_ = 0.1, MCSE_Long_ = 0.1, MCSE_Medium_ = 0, for the rest of the parameters) and each chain took approximately 1 s to converge. The posterior distribution of the Bayesian model showed that the short exposure condition had a mean of 7.41 with a 90% PI [−0.01, 14.85], the medium exposure condition had a mean of 12.16 with 90% PI [1.88, 22.45], and the long exposure condition had a mean of 15.49 with 90% PI [5.2, 25.63], and the Pre-ISQ scores had a positive slope of 1.27 with 90% PI [−1.45, 3.95]. The interaction term between the medium exposure condition and the Pre-ISQ scores had a slope of −1.92 with 90% PI [−6.34, 2.57], and the interaction term between the long exposure condition and the Pre-ISQ scores showed a slope of −2.99 with 90% PI [−8.29, 2.33]. The 90% credible interval of the posterior distributions of the main effects suggests that there is a 38% chance that a difference between the short exposure and medium exposure conditions exist and a 60% chance that a difference between the short exposure and long exposure conditions. See Figure 3 for the observed data and Supplementary Figure 1B for the posterior distributions.

**Figure 4:**
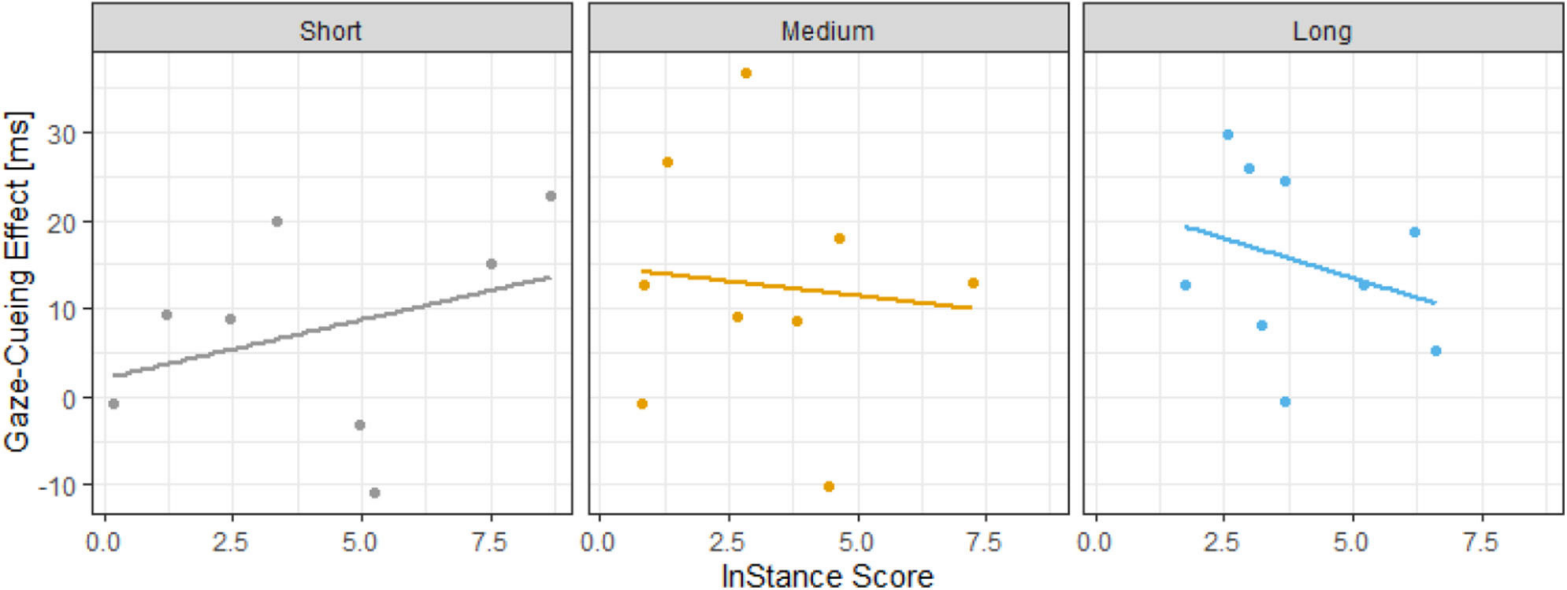
Interaction between exposure and Pre-ISQ scores in GCE. The graph illustrates that an interaction is evident between the length of exposure and Pre-ISQ scores. The interaction shows a positive correlation between ISQ scores and GCE when exposure length is short; however, this relationship becomes more negative as exposure length increases.

Analysis of the interaction effects (i.e., the Pre-ISQ score–GCE relationship as a function of length of exposure), showed that there is a 50% overlap between the 90% credible interval of the short exposure and medium exposure posterior distributions and only a 36% overlap between the 90% credible intervals of the short exposure and long exposure posterior distributions. This suggests that there is a 50% chance that a true difference between short exposure and medium exposure conditions exist, and a 64% chance that a true difference between the short and long exposure conditions exist. These data suggest the linearity of the relationship as it was becoming more negative as we move from the short exposure condition to the long exposure condition. However, the evidence is not very strong, which is possibly due to our limited sample size. See Figure 4 for the observed data and Supplementary Figure 1C for the posterior distributions.

## Discussion

The aim of the current study was to examine if the duration of exposure to a humanoid robot's repetitive behavior in a gaze-cueing task influences adopting the intentional stance toward it, and if adopting of the intentional stance correlates with the GCE depending on the duration of exposure to repetitive behavior. Since previous work suggests that being exposed to robots for long durations decreases the likelihood of anthropomorphizing robots and is associated with negative affective responses, we hypothesized that participants who were exposed to more repetitive behavior would show a decrease in adopting the intentional stance in comparison to those who are exposed to less repetitive robot behavior. Additionally, we expected that the relationship between ISQ scores and GCEs would depend on exposure of the repetitive behavior of iCub (i.e., the relationship changes as a function of being exposed to repetitive gaze behavior of the robot). Finally, this study took a Bayesian approach to investigate these effects, which allows us to examine our effects in terms of probabilities and not whether an effect is significant or not.

To test our hypotheses, we employed a face-to-face gaze-cueing paradigm with iCub, and used an experimental design where observers completed a pre-questionnaire block, then the gaze-cuing experiment with iCub, then a post-questionnaire block. Results of the experiment indicated that the probability of showing a large and positive ISQ change after interacting with iCub was highest for participants who were exposed to less repetitions of iCub's gaze behavior and that the probability of showing a large and positive change decreased as a function of duration exposure. Specifically, the results showed that this probability of showing a positive ISQ difference score decreased by 73% from those who were exposed to iCub for a short duration (i.e., fewer repetitions) to those who were exposed to iCub for a medium duration. This probability decreased even further to 88% from participants who were exposed to iCub in the short exposure condition in comparison to those who were exposed to iCub for longer (i.e., long exposure condition). Interestingly, participants in the long exposure condition did not change their scores after the interaction in respect to their scores prior to interacting with iCub. This indicates that shorter interactions with a robot, even if it shows repetitive behavior, can still have a positive influence on adopting the intentional stance.

Analyses of the GCEs showed that as the length of repetition increased, GCEs were likely to become larger, as short exposure had a high probability of inducing smaller GCEs as compared to the medium exposure condition. This difference between GCEs was even more likely when comparing the short exposure condition and the long exposure condition, which indicates that more trials (i.e., repetitions) are warranted in order to measure GCEs. The results also showed that, as hypothesized, the relationship between adopting the intentional stance and gaze cueing was likely dependent on the length of exposure to repetitive robot behavior. Initially, this relationship was positive, however, as the duration of exposure increased, the relationship showed a tendency to be more negative. While this effect was not very strong, possibly due to being statistically underpowered, it suggests that longer exposures to repetitive robot behavior can reverse the positive relationship between joint attention (a marker of social attunement) and adoption of intentional stance.

The finding that short exposure durations were likely to have a positive effect on subjective ratings of adopting the intentional stance is in line with prior work that used the gaze-cueing paradigm and found that subjective ratings of the mind status of a robot increases after completing the gaze-cueing paradigm (Abubshait and Wiese, 2017; Abubshait et al., 2020b) and other studies showing that initial impressions influenced subjective ratings positively, but latter interactions kept the interactions unchanged (Paetzel et al., 2020). Furthermore, the positive impact of short interactions on adopting the intentional stance might be due to interacting with a real embodied humanoid robot, which initially might evoke social attunement, due to its physical presence (Wainer et al., 2007). However, this effect might diminish after medium and long exposure conditions, due to the repetitiveness of its behavior.

The finding that gaze cueing was likely related to adopting the intentional stance depending on exposure is supported by prior work investigating social engagement between children and social robots that shows that initial interactions with robots are paired with high social engagement but that this social engagement is reduced after a while (Komatsubara et al., 2014; Coninx et al., 2016; Ahmad et al., 2017). This suggests that the reduction in social engagement can generalize to other populations and not simply children. This account is also supported by fMRI studies that argue that the action perception network, a network in the brain that is involved in understanding and predicting others' actions (Decety and Grèzes, 1999), is activated when trying to predict movements from others' gaze behavior (Ramsey et al., 2012) and unfamiliar robotic behavior (Cross et al., 2012). After viewing repetitive actions performed by humans and robots, this activation is reduced for only robotic agents but not humans (Gazzola et al., 2007), which suggests that attention orienting to gaze cues (a phenomenon related to the social brain; Wiese et al., 2018) can be influenced by being exposed to repetitive behaviors.

The results of this study highlight an important issue in the field, which is that many of the effects of attributing intentionality to robots that we see are short-lived. Epley et al. (2007) theory of anthropomorphism provide an explanation to why, which is that when people are exposed to repetitive behaviors from an agent, it can reduce their motivation to understand the agent's behavior, which reduces the propensity to anthropomorphize the agent and adopt the intentional stance toward it. Not only do we find this short-lived effect in our data, but other studies also have found similar results. For example, when using entertainment robots, de Graaf et al. (2015) have found that the most important robot characteristic to be perceived as social is that the robot should be able to verbally respond in a free and social manner (i.e., unpredictable) and not a preprogrammed way.

This result generalizes to other areas in HRI that use repetitive interactions with a robot as interventions with populations diagnosed with autism spectrum condition (ASC). For example, Zheng et al. (2020) examined the effect of repeated exposure to a robotic platform and its effectiveness in improving the joint attention skills of children with ASC. They found that, unlike the suggestion of prior work, learned skills are not maintained over longer periods (Zheng et al., 2020). Similarly, Anzalone et al. (2019) showed a similar pattern where developing children typically did not show improvements in joint attention after prolonged exposures to a robot. These studies show that exposure over longer periods may not have a clear benefit to HRI. The results of our study shed light on this by showing that changes in subjective judgments of intentionality do change when interactions are short-lived. However, after long exposure to iCub's repetitive behavior, the change in attribution of intentionality diminished. Therefore, roboticists have to carefully investigate how longer durations of exposure can influence the human interaction partner.

The current study also illustrates how a Bayesian framework can be a better method in quantifying HRI. HRI studies often have limited sample sizes due to their complex protocols (i.e., sample sizes of about 20 participants; e.g., Mutlu et al., 2006; Terada et al., 2007; Wainer et al., 2007; Wang and Lewis, 2007; Brown and Howard, 2013; de Graaf et al., 2015; Warren et al., 2015; Zheng et al., 2020), which is problematic when analyzing data using parametric statistics. By using a Bayesian framework, we assume that the data are fixed and that the parameter estimates are dynamic, while the opposite is true for parametric methods. In other words, we assume that our parameter estimates for specific behaviors (e.g., gaze-cueing effects in reaction times) follow a distribution as opposed to it having a fixed estimate. By doing so, this method allows us to update our prior knowledge from past studies and estimate an updated parameter distribution. Not only does it allow us to update our knowledge from past studies, but it also allows roboticists to use probabilities of an effect in question. Using probabilities to quantify HRI is much more informative than using significance thresholds. For example, it is more informative for researchers to know which robot has a 70% chance of successfully engaging in joint attention with children with ASC as opposed to which robot significantly engages in joint attention with children with ASC.

Despite the advantages of the methods used here, it is also important to discuss some of the limitations associated with the current study. First, the small sample size could render the results unstable. While this could be a major issue when using parametric analyses due to violations of the general linear model's assumptions, we do not believe that the small sample size dramatically influenced our results. We believe that our results are stable due to using a Bayesian framework, and more so due to using uninformative priors (i.e., uninformative priors are considered conservative even though we could have used informed priors from prior gaze-cueing studies with iCub: e.g., Kompatsiari et al., 2018). By doing so, we inflate our credible intervals, which underestimates the confidence of our results. Another possible limitation is the use of a pre-/post-test design. In this study, participants had to complete a questionnaire that measured their attribution of intentionality to robots prior to completing the gaze-cueing experiment. This design (i.e., participation in the test prior to the experiment) could have biased the participants into attributing intentions to iCub. While this is a limitation that can be addressed by using ecologically valid behavioral measures of intention attribution (e.g., Mwangi et al., 2018), due to our controlled lab setting this method was not feasible. Still, the fact that participants did show group differences when they completed short, medium, and long gaze-cueing tasks with iCub suggests that the length of exposure does influence our interactions with robots in general.

This study provides important contribution regarding implementation of classical experimental protocols in HRI studies by showing two effects that oppose one another. First, our study showed that more trials (i.e., repetitions) were needed in order to measure a social-cognitive process that is associated with social attunement (i.e., gaze cueing), as the gaze-cueing effect was more likely more positive as repetitions increased. On the other hand, subjective ratings of adopting the intentional stance were likely less related to GCEs as repetitions (i.e., trials) increased in the gaze-cueing paradigm, which suggests that studies measuring these two constructs (i.e., social attention and adopting the intentional stance) may sacrifice the validity of one measurement over the other. In our case, the validity of social attention was likely to increase with more trials, but the validity of measuring subjective intention attribution was likely to decrease with more repetitions. Since the data suggest that we might not increase the likelihood of adopting the intentional stance toward an agent when it displays repetitive behavior over a longer duration, it poses a challenge on how to maintain sufficient number of trials for statistical power without decreasing the reception of the robot as an intentional or a human-like agent. While the gaze-cueing paradigm might be successful in evoking joint attention, it might not decrease the perception of the robot as human-like or intentional.

## Data Availability Statement

The datasets presented in this study can be found in online repositories. The names of the repository/repositories and accession number(s) can be found at: https://osf.io/y6c9b/.

## Ethics Statement

The studies involving human participants were reviewed and approved by Comitato Etico Regione Liguria. The patients/participants provided their written informed consent to participate in this study.

## Author Contributions

AA and AW conceptualized the study, interpreted the results, and wrote the manuscript. AA collected and analyzed the data. All authors contributed to the article and approved the submitted version.

## Conflict of Interest

The authors declare that the research was conducted in the absence of any commercial or financial relationships that could be construed as a potential conflict of interest.

## Footnotes

^1^While studies have shown positive outcomes of attributing intentionality to robots in HRI, future studies should consider ethical implications of attributing intentionality toward an inanimate object. For similar arguments (see de Graaf, 2016; Lake et al., 2017).

## References

[B1] Abubshait A. Momen A. Wiese E. (2020a). Pre-exposure to ambiguous faces modulates top-down control of attentional orienting to counterpredictive gaze cues. Front. Psychol. 11:2234 10.3389/fpsyg.2020.02234 33013584 PMC7509110

[B2] Abubshait A. Weis P. P. Wiese E. (2020b). Does context matter? Effects of robot appearance and reliability on social attention differs based on lifelikeness of gaze task. Int. J. Soc. Robot. 10.1007/s12369-020-00675-4. [Epub ahead of print].

[B3] Abubshait A. Wiese E. (2017). You look human, but act like a machine: agent appearance and behavior modulate different aspects of human–robot interaction. Front. Psychol. 8:1393. 10.3389/fpsyg.2017.01393 28878703 PMC5572356

[B4] Admoni H. Bank C. Tan J. Toneva M. Scassellati B. (2011). Robot gaze does not reflexively cue human attention, in Proceedings of the 33rd Annual Conference of the Cognitive Science Society, eds Carlson L. Hölscher C. Shipley T. (Austin, TX: Cognitive Science Society), 1983–1988.

[B5] Ahmad M. I. Mubin O. Orlando J. (2017). Adaptive social robot for sustaining social engagement during long-term children-robot interaction. Int. J. Hum. Comput. Interact. 33, 1–21. 10.1080/10447318.2017.1300750

[B6] Anzalone S. M. Xavier J. Boucenna S. Billeci L. Narzisi A. Muratori F. (2019). Quantifying patterns of joint attention during human-robot interactions: an application for autism spectrum disorder assessment. Pattern Recognit. Lett. 118, 42–50. 10.1016/j.patrec.2018.03.007

[B7] Baron-Cohen S. (1995). Mindblindness: An Essay on Autism and Theory of Mind. eds Gleitman L. Newport E. Spelke E. (Cambridge, MA: MIT Press). 10.7551/mitpress/4635.001.0001

[B8] Bartneck C. (2003). Interacting with an embodied emotional character, in Proceedings of the Design for Pleasurable Products Conference (Pittsburgh, PA), 55–60. 10.1145/782896.782911

[B9] Bergmann K. Eyssel F. Kopp S. (2012). A second chance to make a first impression? How appearance and nonverbal behavior affect perceived warmth and competence of virtual agents over time, in Intelligent Virtual Agents, Vol. 7502, eds Nakano Y. Neff M. Paiva A. Walker M. (Berlin; Heidelberg: Springer), 126–138.

[B10] Breazeal C. Kidd C. Thomaz A. Hoffman G. Berlin M. (2005). Effects of nonverbal communication on efficiency and robustness in human-robot teamwork, in Proceedings of International Conference on Intelligent Robots and Systems (Piscataway, NJ: IEEE), 708–713. 10.1109/IROS.2005.1545011

[B11] Brown L. Howard A. M. (2013). Engaging children inmath education using a socially interactive humanoid robot, in 2013 13th IEEE-RAS International Conference on Humanoid Robots (Humanoids) (Atlanta, GA), 183–188. 10.1109/HUMANOIDS.2013.7029974

[B12] Carter E. J. Mistry M. N. Carr G. P. K. Kelly B. A. Hodgins J. K. (2014). Playing catch with robots: Incorporating social gestures into physical interactions, in Proceedings of the IEEE International Symposium on Robot and Human Interactive Communication (Edinburgh), 231–236. 10.1109/ROMAN.2014.6926258

[B13] Caruana N. de Lissa P. McArthur G. (2017). Beliefs about human agency influence the neural processing of gaze during joint attention. Soc. Neurosci. 12, 194–206. 10.1080/17470919.2016.1160953 26942996

[B14] Ciardo F. Beyer F. De Tommaso D. Wykowska A. (2020). Attribution of intentional agency towards robots reduces one's own sense of agency. Cognition 194:104109. 10.1016/j.cognition.2019.104109 31675616

[B15] Coninx A. Baxter P. Oleari E. Bellini S. Bierman B. Henkemans O. B. (2016). Towards long-term social child-robot interaction: using multi-activity switching to engage young users. J. Hum. Robot Interact. 5, 32–67. 10.5898/JHRI.5.1.Coninx

[B16] Cross E. S. Liepelt R. de C. Hamilton A. F. Parkinson J. Ramsey R.. (2012). Robotic movement preferentially engages the action observation network. Hum. Brain Mapp. 33, 2238–2254. 10.1002/hbm.21361 21898675 PMC6870135

[B17] Cui G. Zhang S. Geng H. (2014). The impact of perceived social power and dangerous context on social attention. PLoS ONE 9:e114077. 10.1371/journal.pone.0114077 25464385 PMC4252089

[B18] Dalmaso M. Galfano G. Coricelli C. Castelli L. (2014). Temporal dynamics underlying the modulation of social status on social attention. PLoS ONE 9:e93139. 10.1371/journal.pone.0093139 24667700 PMC3965511

[B19] de Graaf M. M. A. (2016). An ethical evaluation of human–robot relationships. Int. J. Soc. Robot. 8, 589–598. 10.1007/s12369-016-0368-5

[B20] de Graaf M. M. A. Ben Allouch S. van Dijk J. A. G.M. (2015). What makes robots social?: a user's perspective on characteristics for social human-robot interaction, in Social Robotics, Vol. 9388, eds Tapus A. André E. Martin J.-C. Ferland F. Ammi M. (Paris: Springer International Publishing), 184–193. 10.1007/978-3-319-25554-5_19

[B21] Decety J. Grèzes J. (1999). Neural mechanisms subserving the perception of human actions. Trends Cogn. Sci. 3, 172–178. 10.1016/S1364-6613(99)01312-1 10322473

[B22] Dennett D. C. (1989). The Intentional Stance. Cambridge, MA: MIT Press.

[B23] Dennett D. C. Haugeland J. (1987). Intentionality, in The Oxford Companion to the Mind, eds Gregory R. L. (Oxford University Press). Available online at: http://cogprints.org/252/ (accessed February 3, 2020).

[B24] Driver J. Davis G. Ricciardelli P. Kidd P. Maxwell E. Baron-Cohen S. (1999). Gaze perception triggers reflexive visuospatial orienting. Vis. Cogn. 6, 509–540. 10.1080/135062899394920

[B25] Epley N. Waytz A. Cacioppo J. T. (2007). On seeing human: a three-factor theory of anthropomorphism. Psychol. Rev. 114, 864–886. 10.1037/0033-295X.114.4.864 17907867

[B26] Friesen C. K. Kingstone A. (1998). The eyes have it! Reflexive orienting is triggered by nonpredictive gaze. Psychon. Bull. Rev. 5, 490–495. 10.3758/BF03208827

[B27] Frith C. D. Frith U. (2006). How we predict what other people are going to do. Brain Res. 1079, 36–46. 10.1016/j.brainres.2005.12.126 16513098

[B28] Fussell S. R. Kiesler S. Setlock L. D. Yew V. (2008). How people anthropomorphize robots, in Proceedings of the 3rd ACM/IEEE International Conference on Human Robot Interaction (New York, NY: ACM), 145–152. 10.1145/1349822.1349842

[B29] Gazzola V. Rizzolati G. Wicker B. Keysers C. (2007). The anthropomorphic brain: the mirror neuron system responds to human and robotic actions. Neuroimage 35:1674 10.1016/j.neuroimage.2007.02.003 17395490

[B30] Hungr C. J. Hunt A. R. (2012). Physical self-similarity enhances the gaze-cueing effect. Q. J. Exp. Psychol. 65, 1250–1259. 10.1080/17470218.2012.690769 22670723

[B31] Ito A. Hayakawa S. Terada T. (2004). Why robots need body for mind communication—an attempt of eye-contact between human and robot, in RO-MAN 2004. 13th IEEE InternationalWorkshop on Robot and Human Interactive Communication (IEEE Catalog No.04TH8759), (Kurashiki), 473–478.

[B32] Jones B. C. DeBruine L. M. Main J. C. Little A. C. Welling L. L. M. Feinberg D. R.. (2010). Facial cues of dominance modulate the short-term gaze-cuing effect in human observers. Proc. Biol. Sci. R. Soc. 277, 617–624. 10.1098/rspb.2009.1575 19864283 PMC2842686

[B33] Kiesler S. Powers A. Fussell S. R. Torrey C. (2008). Anthropomorphic interactions with a robot and robot–like agent. Soc. Cogn. 26, 169–181. 10.1521/soco.2008.26.2.169

[B34] Kingstone A. Kachkovski G. Vasilyev D. Kuk M. Welsh T. N. (2019). Mental attribution is not sufficient or necessary to trigger attentional orienting to gaze. Cognition 189, 35–40. 10.1016/j.cognition.2019.03.010 30921692

[B35] Komatsubara T. Shiomi M. Kanda T. Ishiguro H. Hagita N. (2014). Can a social robot help children's understanding of science in classrooms?, in Proceedings of the Second International Conference on Human-Agent Interaction (Tsukuba), 83–90. 10.1145/2658861.2658881

[B36] Kompatsiari K. Ciardo F. Tikhanoff V. Metta G. Wykowska A. (2018). On the role of eye contact in gaze cueing. Sci. Rep. 8:17842. 10.1038/s41598-018-36136-2 30552377 PMC6294791

[B37] Kruschke J. (2014). Doing Bayesian Data Analysis: A Tutorial With R, JAGS, and Stan. Cambridge, MA: Academic Press 10.1016/B978-0-12-405888-0.00008-8

[B38] Lachat F. Conty L. Hugueville L. George N. (2012). Gaze cueing effect in a face-to-face situation. J. Nonverbal Behav. 36, 177–190. 10.1007/s10919-012-0133-x

[B39] Lake B. M. Ullman T. D. Tenenbaum J. B. Gershman S. J. (2017). Building machines that learn and think like people. Behav. Brain Sci. 40:e253. 10.1017/S0140525X16001837 27881212

[B40] Langton S. R. H. Bruce V. (1999). Reflexive visual orienting in response to the social attention of others. Vis. Cogn. 6, 541–567. 10.1080/135062899394939

[B41] Looije R. Neerincx M. A. Cnossen F. (2010). Persuasive robotic assistant for health self-management of older adults: design and evaluation of social behaviors. Int. J. Hum. Comput. Stud. 68, 386–397. 10.1016/j.ijhcs.2009.08.007

[B42] Marchesi S. Ghiglino D. Ciardo F. Perez-Osorio J. Baykara E. Wykowska A. (2019). Do we adopt the intentional stance toward humanoid robots? Front. Psychol. 10:450. 10.3389/fpsyg.2019.00450 30930808 PMC6428708

[B43] Mathôt S. Schreij D. Theeuwes J. (2012). OpenSesame: an open-source, graphical experiment builder for the social sciences. Behav. Res. Methods 44, 314–324. 10.3758/s13428-011-0168-7 22083660 PMC3356517

[B44] McElreath R. (2020). Statistical Rethinking: A Bayesian Course wIth Examples in R and STAN. Boca Raton, FL: CRC Press 10.1201/9780429029608

[B45] McNeish D. (2016). On using bayesian methods to address small sample problems. Struct. Equat. Model. Multidisc. J. 23, 750–773. 10.1080/10705511.2016.1186549

[B46] Metta G. Fitzpatrick P. Natale L. (2006). YARP: Yet another robot platform. Int. J. Adv. Robot. Syst. 3:8 10.5772/5761

[B47] Metta G. Sandini G. Vernon D. Natale L. Nori F. (2008). The iCub humanoid robot: an open platform for research in embodied cognition, in Proceedings of the 8th Workshop on Performance Metrics for Intelligent Systems (Gaithersburg, MD), 50–56. 10.1145/1774674.1774683

[B48] Morewedge C. K. (2009). Negativity bias in attribution of external agency. J. Exp. Psychol. Gen. 138, 535–545. 10.1037/a0016796 19883135

[B49] Mutlu B. Forlizzi J. Hodgins J. (2006). A storytelling robot: Modeling and evaluation of human-like gaze behavior, in 2006 6th IEEE-RAS International Conference on Humanoid. Robots (Genova: IEEE), 1–6.

[B50] Mwangi E. Barakova E. I. Díaz M. Mallofré A. C. Rauterberg M. (2018). Dyadic gaze patterns during child-robot collaborative gameplay in a tutoring interaction, in 2018 27th IEEE International Symposium on Robot and Human Interactive Communication (RO-MAN) (Nanjing), 856–861. 10.1109/ROMAN.2018.8525799

[B51] Neave N. Jackson R. Saxton T. Hönekopp J. (2015). The influence of anthropomorphic tendencies on human hoarding behaviours. Pers. Individ. Dif. 72, 214–219. 10.1016/j.paid.2014.08.041

[B52] Nummenmaa L. Calder A. J. (2009). Neural mechanisms of social attention. Trends Cogn. Sci. 13, 135–143. 10.1016/j.tics.2008.12.006 19223221

[B53] Ohlsen G. van Zoest W. van Vugt M. (2013). Gender and facial dominance in gaze cuing: emotional context matters in the eyes that we follow. PLoS ONE 8:e59471. 10.1371/journal.pone.0059471 23573199 PMC3616071

[B54] Özdem C. Wiese E. Wykowska A. Müller H. Brass M. Van Overwalle F. (2016). Believing androids – fMRI activation in the right temporo-parietal junction is modulated by ascribing intentions to non-human agents. Soc. Neurosci. 12, 582–593. 10.1080/17470919.2016.1207702 27391213

[B55] Paetzel M. Perugia G. Castellano G. (2020). The persistence of first impressions: the effect of repeated interactions on the perception of a social robot, in Proceedings of the 2020 ACM/IEEE International Conference on Human-Robot Interaction (Cambridge), 73–82. 10.1145/3319502.3374786

[B56] Perez-Osorio J. Müller H. J. Wiese E. Wykowska A. (2015). Gaze following is modulated by expectations regarding others' action goals. PLoS ONE 10:e0143614. 10.1371/journal.pone.0143614 26606534 PMC4659552

[B57] Pfeiffer U. J. Timmermans B. Bente G. Vogeley K. Schilbach L. (2011). A non-verbal turing test: differentiating mind from machine in gaze-based social interaction. PLoS ONE 6:e27591. 10.1371/journal.pone.0027591 22096599 PMC3212571

[B58] Quadflieg S. Mason M. F. Macrae C. N. (2004). The owl and the pussycat: gaze cues and visuospatial orienting. Psychon. Bull. Rev. 11, 826–831. 10.3758/BF03196708 15732690

[B59] Ramsey R. Cross E. S. de C. Hamilton A. F. (2012). Predicting others' actions via grasp and gaze: evidence for distinct brain networks. Psychol. Res. 76, 494–502. 10.1007/s00426-011-0393-9 22120203

[B60] Ristic J. Kingstone A. (2005). Taking control of reflexive social attention. Cognition 94, B55–B65. 10.1016/j.cognition.2004.04.005 15617667

[B61] Schellen E. Wykowska A. (2019). Intentional mindset toward robots—open questions and methodological challenges. Front. Robot. AI. 5:139 10.3389/frobt.2018.00139 PMC7805849 33501017

[B62] Schilbach L. Timmermans B. Reddy V. Costall A. Bente G. Schlicht T.. (2013). Toward a second-person neuroscience. Behav. Brain Sci. 36, 393–414. 10.1017/S0140525X12000660 23883742

[B63] Shepherd S. V. Deaner R. O. Platt M. L. (2006). Social status gates social attention in monkeys. Curr. Biol. 16, 119–120. 10.1016/j.cub.2006.02.013 16488858

[B64] Short E. Hart J. Vu M. Scassellati B. (2010). No fair An interaction with a cheating robot, in 2010 5th ACM/IEEE International Conference on Human-Robot Interaction (HRI) (Osaka), 219–226. 10.1109/HRI.2010.5453193

[B65] Süßenbach F. Schönbrodt F. (2014). Not afraid to trust you: trustworthiness moderates gaze cueing but not in highly anxious participants. J. Cogn. Psychol. 26, 1–9. 10.1080/20445911.2014.945457

[B66] Terada K. Shamoto T. Ito A. Mei H. (2007). Reactive movements of non-humanoid robots cause intention attribution in humans, in 2007 IEEE/RSJ International Conference on Intelligent Robots and Systems (San Diego, CA) 3715–3720. 10.1109/IROS.2007.4399429

[B67] Teufel C. Alexis D. M. Clayton N. S. Davis G. (2010). Mental-state attribution drives rapid, reflexive gaze following. Attent. Percept. Psychophys. 72, 695–705. 10.3758/APP.72.3.695 20348576

[B68] Wainer J. Feil-Seifer D. J. Shell D. A. Mataric M. J. (2007). Embodiment and human-robot interaction: a task-based perspective, in RO-MAN 2007- The 16th IEEE International Symposium on Robot and Human Interactive Communication (Jeju), 872–877. 10.1109/ROMAN.2007.4415207

[B69] Wang J. Lewis M. (2007). Human control for cooperating robot teams. In 2007 2nd ACM/IEEE International Conference on Human-Robot Interaction (HRI) (Arlington, VA: IEEE), 9–16. 10.1145/1228716.1228719

[B70] Warren Z. E. Zheng Z. Swanson A. R. Bekele E. Zhang L. Crittendon J. A.. (2015). Can robotic interaction improve joint attention skills? J. Autism Dev. Disord. 45, 3726–3734. 10.1007/s10803-013-1918-4 24014194 PMC3949684

[B71] Waytz A. Gray K. Epley N. Wegner D. M. (2010). Causes and consequences of mind perception. Trends Cogn. Sci. 14, 383–388. 10.1016/j.tics.2010.05.006 20579932

[B72] Weisman K. Dweck C. S. Markman E. M. (2017). Rethinking people's conceptions of mental life. Proc. Natl. Acad. Sci. U.S.A. 114, 11374–11379. 10.1073/pnas.1704347114 29073059 PMC5664492

[B73] Wiese E. Buzzell G. A. Abubshait A. Beatty P. J. (2018). Seeing minds in others: mind perception modulates low-level social-cognitive performance and relates to ventromedial prefrontal structures. Cogn. Affect. Behav. Neurosci. 18, 837–856. 10.3758/s13415-018-0608-2 29992485

[B74] Wiese E. Metta G. Wykowska A. (2017). Robots as intentional agents: using neuroscientific methods to make robots appear more social. Front. Psychol. 8:1663. 10.3389/fpsyg.2017.01663 29046651 PMC5632653

[B75] Wiese E. Wykowska A. Zwickel J. Müller H. J. (2012). I see what you mean: how attentional selection is shaped by ascribing intentions to others. PLoS ONE 7:e45391. 10.1371/journal.pone.0045391 23049794 PMC3458834

[B76] Woods S. Dautenhahn K. Kaouri C. (2005). Is someone watching me? Consideration of social facilitation effects in human-robot interaction experiments, in Proceedings of 2015 IEEE International Symposium on Computational Intelligence in Robotics and Automation (Espoo), 53–60. 10.1109/CIRA.2005.1554254

[B77] Wykowska A. Wiese E. Prosser A. Müller H. J. (2014). Beliefs about the minds of others influence how we process sensory information. PLoS ONE 9:e94339. 10.1371/journal.pone.0094339 24714419 PMC3979768

[B78] Yamazaki A. Yamazaki K. Burdelski M. Kuno Y. Fukushima M. (2010). Coordination of verbal and non-verbal actions in human–robot interaction at museums and exhibitions. J. Pragmat. 42, 2398–2414. 10.1016/j.pragma.2009.12.023

[B79] Yonezawa T. Yamazoe H. Utsumi A. Abe S. (2007). Gaze-communicative behavior of stuffed-toy robot with joint attention and eye contact based on ambient gaze-tracking, in Proceedings of the 9th International Conference on Multimodal Interfaces (Nagoya Aichi), 140–145. 10.1145/1322192.1322218

[B80] Zheng Z. Nie G. Swanson A. Weitlauf A. Warren Z. Sarkar N. (2020). A randomized controlled trial of an intelligent robotic response to joint attention intervention system. J. Autism Dev. Disord. 50, 2819–2831. 10.1007/s10803-020-04388-5 32026173

