# Supplementary material for: Repetitive Robot Behavior Impacts Perception of Intentionality and Gaze-Related Attentional Orienting
Source: Front Robot AI. 2020 Nov 9;7:565825. doi: 10.3389/frobt.2020.565825 (PMC7805881; doi:10.3389/frobt.2020.565825)
Supplement: Supplementary file 1 [file Data_Sheet_1.PDF]

**Supplementary materials**

**Exploratory questionnaires.** The anthropomorphism questionnaire was divided into two subscales. A general anthropomorphism subscale which measures the tendency to attribute human thoughts, feelings, and motivations to nonhuman objects and a child anthropomorphism questionnaire that measures childhood anthropomorphism tendencies. All items were scored on a 7-point Likert-scale with 0 indicating "Not at all" and 6 being "Very much so". Scores for each subscale were calculated by averaging the items that belonged to each respective subscale, with higher scores indicating more anthropomorphism tendencies.

The Body, Heart, and Mind questionnaire is a three-subscale questionnaire and was administered once at the beginning of the experiment and once at the end. All items were asked in regard to iCub (e.g., "how likely does iCub have a mind?"). Each subscale of the questionnaire measures which facet of the mental is attributed to iCub and to what extent they were attributed. The Body subscale measures an agent's capacity to experience physiological sensations. The Heart subscale measures an agent's capacity to experience social emotions, and the Mind subscale measures an agent's capacity to experience perceptual abilities. All items were recorded using a 7-point Likert-scale, with 0 being "Not capable at all" and 6 being "Highly capable". All subscales were scored individually by averaging the items of each subscale. Higher scores signified more perception of mental capacities.

Finally, the perceived predictivity question was asked twice (i.e., at the beginning and the end of the experiment). The question asked, "Please rate how much you can predict iCub's behavior". The question was recorded using a slider and measured and a 10-point magnitude scale with 0 being "highly unlikely" and 10 being "highly likely".

**Exploratory results.** Exploratory analyses examining the effects of attributed mental capacities and perceived predictivity on Gaze-cueing effects (GCE) showed no relationship between GCE and perceived predictivity ($r(24) = .09$, 90% [-.24, .4]), the heart capacity

(*r*(24) = .03, 90% [-.35, .3]), the body capacity (*r*(24) = -.07, 90% [-.39, .26]), or the Mind capacity (*r*(24) = .3, 90% [-.03, .57]).

Correlations between individual differences in anthropomorphism and adopting the intentional stance showed no effect between ISQ scores and the child anthropomorphism subscale (*r*(24) = .02, 90% [-.31, .34]), or the general anthropomorphism subscale (*r*(24) = .001, 90% [-.32, .33]). found. Correlations between attribution of mental capacities and adopting the intentional stance showed an effect between ISQ scores and the Heart capacity (*r*(24) = .61, 90% [.34, .78]), the Body capacity (*r*(24) = .64, 90% [.39, .80]), and the Mind capacity (*r*(24) = .47, 90% [.17, .69]). Interestingly, we found that the Mind capacity was scored higher overall as compared to the Body capacity or the Heart capacity; see **Figure S2**. We also examined if perceived predictability and attribution of mental capacities ratings changed as a function of exposure and the change in Engagement ratings overtime. All analyses revealed no meaningful effects.

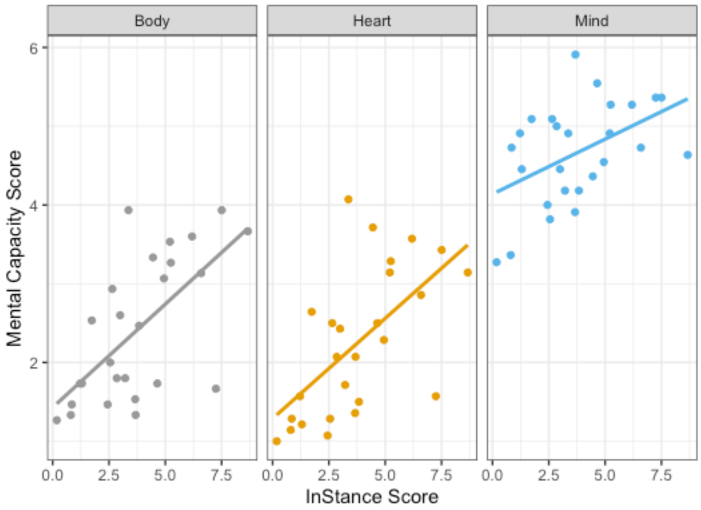


**Figure S1.** Correlations between mental capacities and ISQ scores: the graphs illustrate that attributed mental states are positively correlated with the InStance scores. Also, it seems that overall the Mind capacity had higher overall scores in comparison to the Body capacity and the Heart capacity.

To examine if perceived predictability and attribution of mental capacities ratings changed as a function of exposure, we used an OLS regression for each outcome variable

(i.e., perceived predictability, attributed body capacity, heart capacity, and mind capacity) with the exposure length as a dummy coded predictor variable and each respective pre-questionnaire score as a covariate. The regression model predicting difference scores in perceived predictability showed no strong differences between the short and medium exposure conditions (*b* = -.6, 90% [-2.6, 1.43]), or the short and long exposure conditions (*b* = -.56, 90% [-2.63, 1.49]) in terms of difference perceived predictability scores. Similarly, the regression model predicting the attributed Heart capacity showed no differences between the short and medium exposure conditions (*b* = .21, 90% [-.07, .49]), or the short and long exposure conditions (*b* = -.07, 90% [-.35, .2]) in Heart capacity difference scores. The attributed Body capacity also showed no meaningful differences between the short and medium exposure conditions (*b* = .13, 90% [-.22, .48]), or the short and long exposure conditions (*b* = -.16, 90% [-.5, .16]) in attributed Body capacity difference scores. Finally, the attributed Mind capacity also showed minor differences between the short and medium exposure conditions (*b* = .21, 90% [-.12, .55]), or the short and long exposure conditions (*b* = -.13, 90% [-.47, .2]) in mind difference capacity scores.

To examine the change in Engagement ratings overtime, we used a linear mixed model predicting subjective ratings of engagement from Exposure condition (i.e., a dummy coded variable), Block Number (i.e., 1-16), and their interaction. We also added a random effect to allow variations in both intercepts and slopes for each subject overtime. The linear mixed model revealed a significant intercept (*b* = 6.24, *t*(26.35) = 9.09, p < .001), but no effects for the medium exposure condition (*b* = 1.11, *t*(24.99) = 1.11, p = .25), the long exposure condition (*b* = .01, *t*(24.56) = .01, p = .98), Block number (*b* < -.01, *t*(83.62) = -.08, p = .93), the medium exposure by Block Number interaction (*b* = -.11, *t*(52.14) = -1.24, p = .21), or the long exposure by Block Number interaction (*b* = -.08, *t*(48.36) = -.94, p = .34).

**Discussion of exploratory findings.** One noteworthy finding of the exploratory analyses was that different facets of understanding mental capacities (i.e., Body, Heart, Mind) were related differently to mental capacities towards iCub. Specifically, we found that people generally attribute more perceptual abilities (i.e., Mind capacity) to iCub than physiological sensations (i.e., Body) or emotions (i.e., Heart). This suggests that when designing robots that should evoke the intentional stance, we should consider equipping these robots with characteristics that allow them to be perceived as capable of experiencing perceptual abilities such as making choices, detecting odors, and recognizing others (Weisman et al., 2017). Another finding, albeit unsurprising, is that the three the mental capacities were positively related to adopting the intentional stance. This is unsurprising, as intention attribution and mental capacities are closely related concepts. The finding of this exploratory analysis invites future work to investigate this further.
